# Long-term safety and efficacy of sepetaprost, with or without timolol, in patients with open-angle glaucoma or ocular hypertension: results from the randomised, phase 3 ANGEL-J2 study

**DOI:** 10.1007/s10384-026-01351-2

**Published:** 2026-06-16

**Authors:** Masaru Inatani, Yuko Tone, Toshihiro Ikeda, Raymund Angeles

**Affiliations:** 1 Department of Ophthalmology, Faculty of Medical Sciences, University of Fukui, Yoshida, Japan; 2 Santen Pharmaceutical Co., Ltd., Osaka, Japan; 3 Santen Inc., 2100 Powell Street, Suite 800, Emeryville, CA 94608 USA

**Keywords:** Dual FP/EP3 receptor agonist, Intraocular pressure, Ocular hypertension, Open-angle glaucoma, Sepetaprost

## Abstract

**Purpose:**

To evaluate the long-term safety and intraocular pressure (IOP)-lowering efficacy of sepetaprost, a novel dual FP/EP3 receptor agonist, in patients with open-angle glaucoma (OAG) or ocular hypertension (OHT).

**Study design:**

ANGEL-J2 (NCT05503901) was a phase 3, randomised, parallel-group, open-label study across 20 sites in Japan.

**Methods:**

Adult patients with IOP ≥16 and <22 mmHg were assigned to once-daily sepetaprost 0.002% (Group 1), whereas patients with IOP ≥22 and ≤34 mmHg were randomised 1:1 to once-daily sepetaprost 0.002% alone (Group 2) and with twice-daily timolol 0.5% (Group 3). Over 52 weeks of treatment, efficacy was assessed as the change in mean diurnal IOP from baseline; safety outcomes included the incidence of adverse drug reactions (ADRs).

**Results:**

In total, 131 patients were assigned to Group 1 (n=49), Group 2 (n=42), or Group 3 (n=40). In each group, treatment was associated with rapid and sustained reductions in IOP versus baseline (*p*<0.0001 at all time-points). Mean (± standard error) change in diurnal IOP from baseline at week 52 was −4.40±0.24, −6.38±0.32, and −7.51±0.29 mmHg in Groups 1, 2, and 3, respectively; similarly, mixed model for repeated measures analyses found that reductions in mean diurnal IOP over 52 weeks were greater in Group 3 versus Group 2. ADRs were reported in 80 patients overall (59.2–65.0% across groups), most commonly mild eyelash growth (22.4–42.5%) and mild conjunctival hyperaemia (28.6–30.6%).

**Conclusion:**

Sepetaprost, with or without timolol, demonstrated good tolerability and may represent an efficacious long-term treatment option for OAG or OHT.

**Supplementary Information:**

The online version contains supplementary material available at 10.1007/s10384-026-01351-2.

## Introduction

Lowering intraocular pressure (IOP) remains the only evidence-based treatment strategy for glaucoma [1–3], a leading cause of irreversible vision loss worldwide [4–7]. Several topical IOP-lowering therapies are available for the management of glaucoma, including prostaglandin F (FP) receptor agonists (e.g., latanoprost), prostaglandin E_2_ receptor EP2 subtype (EP2) agonists (e.g., omidenepag isopropyl), β-adrenergic blockers (e.g., timolol), α-adrenergic agonists (e.g., brimonidine), carbonic anhydrase inhibitors (e.g., dorzolamide), and Rho kinase inhibitors (e.g., netarsudil). Current clinical guidelines recommend switching treatments as needed to reduce IOP [5–7]; however, many patients are unable to achieve target IOP levels with existing monotherapies [8]. Multi-drug combination therapy is a subsequent treatment option for these patients [5–7], but the effectiveness of this strategy is often hampered by complex treatment regimens and poor medication adherence [9].

While most prostaglandin analogues for glaucoma are FP receptor agonists, newer agents with alternative mechanisms of action (e.g., omidenepag isopropyl) have been developed with demonstrable efficacy and tolerability [10–13]. Preclinical evidence suggests that the IOP-lowering effects of FP receptor agonists are mediated in part by prostaglandin E_2_ receptor EP3 subtype (EP3) receptors [14–17]; therefore, dual FP/EP3 pathway activation may be a promising strategy to achieve potent IOP-lowering efficacy.

Sepetaprost was developed as a novel dual FP/EP3 receptor agonist [18], and recently received manufacturing and marketing approval in Japan for the treatment of glaucoma and ocular hypertension (OHT) [19]. Several clinical trials demonstrate the safety and efficacy of sepetaprost [20–27]; in particular, the phase 2b ANGEL-2 study shows that once-daily sepetaprost was non-inferior to twice-daily timolol in reducing IOP over 3 months of treatment, and was well tolerated in patients with primary open-angle glaucoma (POAG) or OHT [26]. The recent phase 3 ANGEL-J1 study similarly found that the IOP-lowering efficacy of sepetaprost in POAG or OHT was non-inferior to latanoprost at week 4 and maintained over 3 months [27]. Following on from ANGEL-2 and in parallel with ANGEL-J1, the present ANGEL-J2 study was conducted to assess the longer-term safety and efficacy of sepetaprost, alone or in combination with timolol, in patients with open-angle glaucoma (OAG) or OHT.

## Patients and methods

### Study design

ANGEL-J2 was a phase 3, multicentre, randomised, parallel-group, open-label study of sepetaprost, as monotherapy or in combination with timolol, in patients with OAG or OHT. The study was conducted at 20 clinical sites in Japan from August 2022 to January 2024. The conduct of the study was compliant with the Declaration of Helsinki, the Act on Securing Quality, Efficacy and Safety of Products Including Pharmaceuticals and Medical Devices (PMD Act), and Japanese Ministerial Ordinance on Good Clinical Practice. The ANGEL-J2 study protocol was reviewed and approved by the Institutional Review Boards of the participating sites, and all patients provided written informed consent. ANGEL-J2 is registered with ClinicalTrials.gov (NCT05503901) and the Japanese Registry of Clinical Trials (jRCT2031220266).

### Study population

The study included adults aged ≥ 18 years with OAG (including POAG or normal tension glaucoma [NTG]), pseudoexfoliation glaucoma, pigmentary glaucoma, or OHT in both eyes. Eligible patients were also required to have a corrected visual acuity (VA) of +0.60 logarithm of the minimum angle of resolution (logMAR) units or better (decimal VA ≥ 0.3), a central corneal thickness of 480–620 µm, and an anterior chamber angle of Shaffer grade ≥ 2 in both eyes. At the baseline study visit (described below), IOP at three time points (9:00, 13:00, and 17:00) was required to be ≥ 16 mmHg in at least one eye and ≤ 34 mmHg in both. Patients were excluded if they had any prior history of IOP-lowering ocular surgery; received any ocular surgery or laser treatment within 90 days of screening or throughout the study period; or initiated, discontinued, or changed any topical or systemic treatment that may affect IOP (excluding IOP-lowering treatments for glaucoma or OHT) within 30 days of screening. Full inclusion and exclusion criteria are available upon request.

One eye per patient was designated the study eye. The study eye was selected as the eye with the higher IOP at baseline; if baseline IOP was the same in both eyes, the right eye was selected.

### Study procedures

**Fig. 1:**
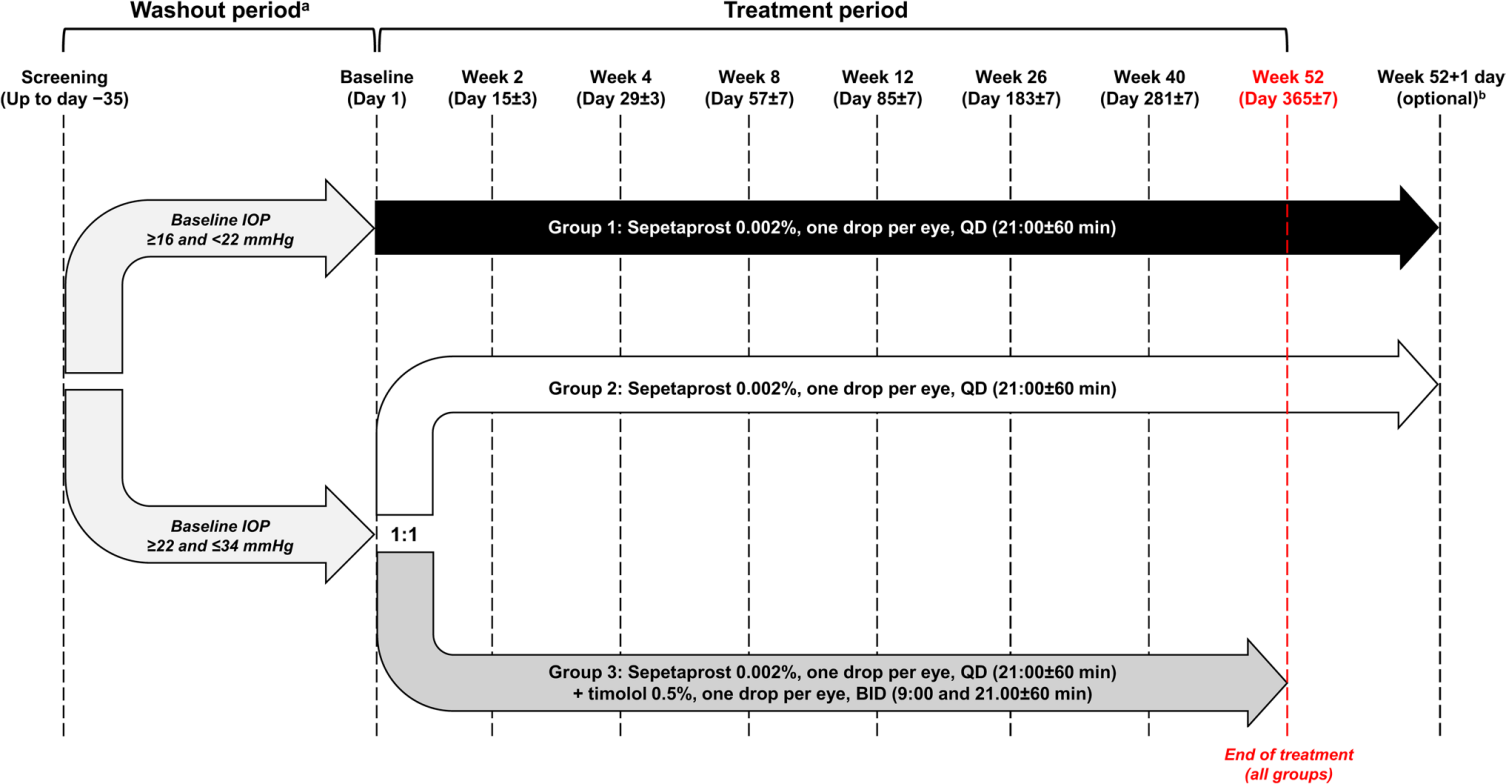
ANGEL-J2 study design. ^a^During the washout period, eligible patients discontinued all prior IOP-lowering treatments. The duration of the washout period for each patient was based on the prior treatment(s) received, plus up to 7 additional days (up to 35 days in total): 7 days for miotics and carbonic anhydrase inhibitors; 14 days for α-adrenergic agonists and α/β-adrenergic agonists; and 28 days for α-adrenergic antagonists, β-adrenergic blockers, prostaglandin analogues, and Rho kinase inhibitors. For patients receiving multiple prior treatments, the longest washout period was applied; treatment-naïve patients could progress straight to the next study visit without the need for a washout period. ^b^At the week 52 study visit, patients in Groups 1 and 2 were invited to attend an optional final study visit at week 52+1 day (approximately 36–44 hours after the last dose of sepetaprost). *BID* twice daily, *IOP* intraocular pressure, *QD* once daily

After screening, eligible patients entered a washout period of ≤ 35 days, depending on the previous IOP-lowering treatment(s) received (Fig. 1). Washout was followed by a 52-week treatment period, where patients with a baseline IOP ≥ 16 and < 22 mmHg (i.e., those likely to receive IOP-lowering monotherapy in clinical practice) were assigned to Group 1, and patients with a baseline IOP ≥ 22 and ≤ 34 mmHg (i.e., those likely to receive IOP-lowering combination therapy in clinical practice) were randomised 1:1 into Groups 2 and 3. Patients in Groups 2 and 3 were randomised using the permutation block method (block size 4), generated by the treatment allocation manager using a central computer system. The code used for treatment allocation was sealed and stored. Throughout the study, IOP was calculated as the mean of two consecutive IOP measurements at each time point; if the two measurements differed by ≥ 3 mmHg, a third measurement was taken and IOP was calculated as the median.

Patients assigned to Groups 1 and 2 were instructed to apply one drop of sepetaprost 0.002% ophthalmic solution OU, once daily at 21:00 (± 60 minutes; Fig. 1). Group 3 also administered sepetaprost 0.002% as described above, but additionally applied one drop of timolol 0.5% ophthalmic solution OU, twice daily at 9:00 and 21:00 (± 60 minutes). At 21:00 each night, patients in Group 3 were instructed to apply sepetaprost before timolol, with an interval of ≥ 5 minutes in between. All patients started their assigned treatment on the night of the baseline study visit; patients in Groups 1 and 2 completed their last dose (of sepetaprost) on the night before the week 52 study visit, and those in Group 3 completed their last dose (of timolol) at 9:00 on the day of the week 52 visit.

During the treatment period, all patients attended follow-up visits at weeks 2, 4, 8, 12, 26, 40, and 52 (Fig. 1). Ocular assessments at each visit included VA tests; slit-lamp microscopy; frontal and lateral photographs of each eye (including the iris, eyelashes, and eyelids); and tonometry to measure IOP at 9:00, 13:00, and 17:00 (all ± 60 minutes). On the morning of each study visit, patients in Group 3 applied their 9:00 dose of timolol after their 9:00 IOP measurement. At the week 52 study visit, patients in Groups 1 and 2 were invited to attend an optional final study visit at week 52+1 day (approximately 36–44 hours after the last dose of sepetaprost), where IOP and other key ocular assessments were performed. Adverse events (AEs) were monitored throughout the study and coded using Medical Dictionary of Regulatory Activities thesaurus terms (MedDRA version 25.0).

### Outcome measures

Efficacy was assessed as the change in mean diurnal IOP from baseline over 52 weeks of treatment, plus at week 52+1 day in Groups 1 and 2. Mean diurnal IOP was calculated as the mean of the 9:00, 13:00, and 17:00 IOP measurements in the study eyes at each visit. Safety outcomes included the incidence and severity of AEs and adverse drug reactions (ADRs; defined as any AE related to study treatment), as well as the incidence of ocular ADRs by time of onset in Groups 1 and 2.

### Statistical analysis

To ensure that ≥ 100 patients completed the 52-week treatment period (in line with International Council for Harmonisation E1 guidelines on population exposure [28]), the target sample size for this study was 138 patients (46 patients per treatment group). Baseline characteristics and treatment efficacy were evaluated in the full analysis set, defined as all patients who received ≥ 1 dose of their assigned study treatment, and who had evaluable IOP data at baseline and ≥ 1 post-baseline study visit. Baseline data and mean diurnal IOP over time were summarised using descriptive statistics, including mean, standard deviation (SD), standard error (SE), median, and range for continuous variables; and the number and proportion of patients for categorical variables. Analyses were based on observed data, with no imputation for missing values.

Change in mean diurnal IOP from baseline over time was evaluated for each treatment group using paired t-tests, and additional mixed model for repeated measures (MMRM) analyses were performed to compare changes in mean diurnal IOP between Groups 2 and 3. MMRM analyses included treatment, visit, and treatment-by-visit interaction as fixed effects, baseline IOP as a covariate, and subject as a random effect; within-subject errors were modelled using an unstructured covariance matrix. MMRM data were summarised using least-squares (LS) means, SEs, differences in LS means between treatment groups, and associated 95% confidence intervals (CI).

Safety outcomes were evaluated in the safety analysis set, defined as all patients who received ≥1 dose of their assigned study treatment. Safety was assessed through descriptive summaries of AEs and ADRs reported throughout the study period. Statistical analyses were performed using SAS version 9.4 or later (SAS Institute, Cary, NC, USA).

## Results

### Study population

In total, 172 patients with OAG or OHT gave informed consent to participate in the ANGEL-J2 study (Supplementary Fig. 1). After exclusions, 131 patients were enrolled, including 49 patients with baseline IOP ≥ 16 and < 22 mmHg (cohort 1) and 82 patients with baseline IOP ≥ 22 and ≤ 34 mmHg (cohort 2). All 49 patients in cohort 1 were assigned to sepetaprost (Group 1), and patients in cohort 2 were randomised to sepetaprost (Group 2; n=42) or sepetaprost plus timolol (Group 3; n=40). All 131 patients enrolled in the study were included in the full and safety analysis sets to evaluate efficacy and safety outcomes, respectively.

At baseline, the mean±SD age of the full analysis set was 63.8±12.7 years, 46.6% of patients were women, and 48.1% had a primary diagnosis of POAG (Table 1). By design, patients in Group 1 had lower mean diurnal IOP at baseline versus those in Groups 2 and 3 (18.8 mmHg vs. 23.2–24.0 mmHg, respectively). Other baseline characteristics were generally balanced across treatment groups, and there were no major differences between Groups 2 and 3.

**Table 1:** Baseline patient demographics and clinical characteristics (full analysis set)

	Group 1: Sepetaprost (n=49)	Group 2: Sepetaprost (n=42)	Group 3: Sepetaprost + timolol (n=40)	Group 1 + 2: Sepetaprost (n=91)	Total (N=131)
*Age, years*					
Mean±SD	64.7±11.5	62.1±14.6	64.7±12.0	63.5±13.0	63.8±12.7
Median (range)	68.0 (21, 79)	65.0 (27, 83)	68.0 (28, 84)	67.0 (21, 83)	67.0 (21, 84)
<65, n (%)	20 (40.8)	19 (45.2)	18 (45.0)	39 (42.9)	57 (43.5)
≥65, n (%)	29 (59.2)	23 (54.8)	22 (55.0)	52 (57.1)	74 (56.5)
*Female sex, n (%)*	23 (46.9)	21 (50.0)	17 (42.5)	44 (48.4)	61 (46.6)
*Primary diagnosis, n (%)*					
NTG	16 (32.7)	2 (4.8)	1 (2.5)	18 (19.8)	19 (14.5)
OHT	11 (22.4)	19 (45.2)	19 (47.5)	30 (33.0)	49 (37.4)
POAG	22 (44.9)	21 (50.0)	20 (50.0)	43 (47.3)	63 (48.1)
*Mean diurnal IOP, mmHg*					
Mean±SD	18.8±1.4	23.2±1.5	24.0±1.8	20.9±2.6	21.8±2.8
Median (range)	18.8 (16.0, 21.8)	23.0 (22.0, 30.0)	23.5 (22.2, 30.0)	20.5 (16.0, 30.0)	22.3 (16.0, 30.0)
*IOP at 9:00, mmHg*					
Mean±SD	19.5±1.9	23.5±2.0	24.2±2.3	21.4±2.8	22.3±3.0
Median (range)	19.5 (16.0, 23.5)	23.0 (20.0, 31.5)	24.0 (21.5, 31.5)	22.0 (16.0, 31.5)	22.5 (16.0, 31.5)
*IOP at 13:00, mmHg*					
Mean±SD	18.7±1.6	23.2±1.6	24.2±2.1	20.8±2.8	21.8±3.1
Median (range)	19.0 (16.0, 22.0)	23.0 (21.5, 30.5)	23.8 (21.0, 31.0)	21.0 (16.0, 30.5)	22.0 (16.0, 31.0)
*IOP at 17:00, mmHg*					
Mean±SD	18.3±1.5	22.9±1.5	23.6±1.8	20.4±2.7	21.4±2.9
Median (range)	18.0 (16.0, 22.0)	23.0 (20.0, 28.0)	23.0 (20.0, 30.0)	20.0 (16.0, 28.0)	22.0 (16.0, 30.0)
*CCT, µm*					
Mean±SD	540.0±33.2	549.2±29.0	550.6±31.6	544.3±31.5	546.2±31.5
Median (range)	533.0 (466, 619)	549.0 (489, 623)	555.5 (494, 610)	543.0 (466, 623)	547.0 (466, 623)
*VA, LogMAR units*					
Mean±SD	−0.06±0.08	−0.05±0.09	−0.05±0.07	−0.06±0.08	−0.05±0.08
Median (range)	−0.08 (−0.18, 0.15)	−0.08 (−0.30, 0.15)	−0.08 (−0.18, 0.10)	−0.08 (−0.30, 0.15)	−0.08 (−0.30, 0.15)
*Degree of angle closure, n (%)*					
Shaffer grade 2	2 (4.1)	1 (2.4)	1 (2.5)	3 (3.3)	4 (3.1)
Shaffer grade 3–4	47 (95.9)	41 (97.6)	39 (97.5)	88 (96.7)	127 (96.9)

Group 1 included patients with baseline IOP ≥16 and <22 mmHg; Groups 2 and 3 included patients with baseline IOP ≥22 and ≤34 mmHg

*CCT* central corneal thickness, *IOP* intraocular pressure, *LogMAR* logarithm of the minimum angle of resolution, *NTG* normal tension glaucoma, *OHT* ocular hypertension, *POAG* primary open-angle glaucoma, *SD* standard deviation, *VA* visual acuity

### Efficacy outcomes

**Fig. 2:**
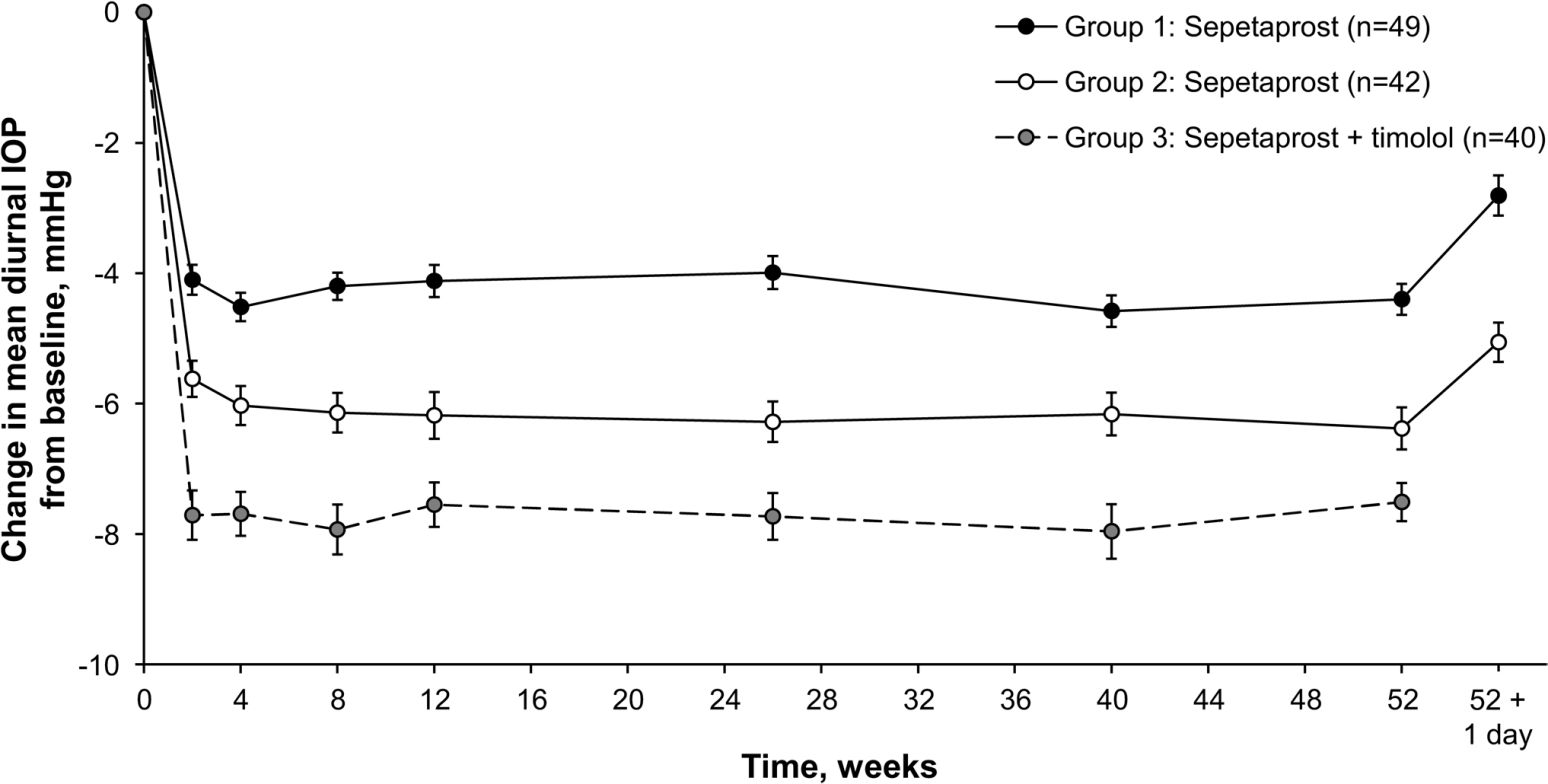
Change in mean diurnal IOP from baseline over time (full analysis set). Data are mean ± standard error. Group 1 included patients with baseline IOP ≥16 and <22 mmHg; Groups 2 and 3 included patients with baseline IOP ≥22 and ≤34 mmHg. Consenting patients in Group 1 (n=32) and Group 2 (n=33) attended an optional final study visit at week 52+1 day, approximately 36–44 hours after the last dose of sepetaprost. For all treatment groups, change in mean diurnal IOP was statistically significant at all time points versus baseline (all *p*<0.0001, based on paired t-tests). *IOP* intraocular pressure

Sepetaprost, as monotherapy or in combination with timolol, was associated with significant and sustained reductions in mean diurnal IOP over 52 weeks of treatment (*p*<0.0001 versus baseline for all treatment groups and time points; Fig. 2). In Group 1, the mean±SE change in mean diurnal IOP from baseline was maintained from week 2 (−4.10±0.23 mmHg) through to week 52 (−4.40±0.24 mmHg). Reductions in mean diurnal IOP from baseline were comparatively greater in Group 2 (−5.62±0.28 mmHg at week 2 and −6.38±0.32 mmHg at week 52) and further improved in Group 3 (−7.71±0.38 mmHg and −7.51±0.29 mmHg, respectively).

MMRM analyses supported the additive IOP-lowering effects of adding timolol to sepetaprost (Table 2). In Group 3, LS mean reductions in mean diurnal IOP from baseline were significantly greater than Group 2 at each time point from week 2 to 40 (all *p*<0.05), and numerically greater than Group 2 at week 52. Among patients in Group 1 (n=32) and Group 2 (n=33) who attended a final study visit at week 52+1 day (approximately 36–44 hours after the last dose of sepetaprost), mean±SE change in mean diurnal IOP from baseline was less than that observed at week 52 (−2.81±0.31 mmHg and −5.06±0.30 mmHg, respectively), but remained significantly reduced versus baseline (both *p*<0.0001; Fig. 2).

**Table 2:** MMRM analysis of change in mean diurnal IOP from baseline over time (full analysis set)

Change in mean diurnal IOP from baseline, mmHg	Group 2: Sepetaprost (n=42)	Group 3: Sepetaprost + timolol (n=40)
*Week 2*		
LS mean±SE	−5.85±0.29	−7.48±0.30
Difference±SE	1.62±0.42	
95% CI	0.79, 2.46	
*Week 4*		
LS mean±SE	−6.26±0.28	−7.47±0.28
Difference±SE	1.21±0.40	
95% CI	0.42, 2.01	
*Week 8*		
LS mean±SE	−6.37±0.29	−7.71±0.30
Difference±SE	1.34±0.42	
95% CI	0.50, 2.19	
*Week 12*		
LS mean±SE	−6.45±0.29	−7.33±0.30
Difference±SE	0.88±0.42	
95% CI	0.05, 1.72	
*Week 26*		
LS mean±SE	−6.54±0.29	−7.50±0.30
Difference±SE	0.96±0.42	
95% CI	0.13, 1.79	
*Week 40*		
LS mean±SE	−6.33±0.31	−7.77±0.32
Difference±SE	1.45±0.45	
95% CI	0.55, 2.35	
*Week 52*		
LS mean±SE	−6.56±0.28	−7.31±0.28
Difference±SE	0.75±0.40	
95% CI	−0.05, 1.54	

Data are based on MMRM analyses, which included treatment, visit, and treatment-by-visit interaction as fixed effects, baseline IOP as a covariate, and subject as a random effect. Within-subject errors were modelled using an unstructured covariance matrix

*CI* confidence interval, *IOP* intraocular pressure, *LS* least-squares, *MMRM* mixed model for repeated measures, *SE* standard error

### Safety outcomes

Overall, the incidence and severity of AEs and ADRs were comparable between treatment groups (Table 3). All AEs except one were mild or moderate in severity; one severe AE of pneumonia was reported in 1 patient (2.5%) in Group 3, but was not related to study treatment. Serious AEs were reported in 3 (6.1%), 2 (4.8%), and 3 patients (7.5%) in Groups 1, 2, and 3, respectively; however, none were treatment related.

**Table 3:** Summary of AEs and ADRs during the treatment period (safety analysis set)

n (%)	Group 1: Sepetaprost (n=49)	Group 2: Sepetaprost (n=42)	Group 3: Sepetaprost + timolol (n=40)	Group 1 + 2: Sepetaprost (n=91)	Total (N=131)
*Patients with any event*					
AE	43 (87.8)	32 (76.2)	38 (95.0)	75 (82.4)	113 (86.3)
Mild	39 (79.6)	28 (66.7)	33 (82.5)	67 (73.6)	100 (76.3)
Moderate	4 (8.2)	4 (9.5)	4 (10.0)	8 (8.8)	12 (9.2)
Severe	0	0	1 (2.5)	0	1 (0.8)
SAE	3 (6.1)	2 (4.8)	3 (7.5)	5 (5.5)	8 (6.1)
ADR	29 (59.2)	25 (59.5)	26 (65.0)	54 (59.3)	80 (61.1)
Ocular ADR	28 (57.1)	25 (59.5)	26 (65.0)	53 (58.2)	79 (60.3)
Non-ocular ADR	2 (4.1)	0	0	2 (2.2)	2 (1.5)
Serious ADR	0	0	0	0	0
AE leading to treatment discontinuation	3 (6.1)	3 (7.1)	2 (5.0)	6 (6.6)	8 (6.1)
AE resulting in death	0	0	0	0	0
*ADRs with overall incidence ≥2%*^*a*^					
Growth of eyelashes	11 (22.4)	15 (35.7)	17 (42.5)	26 (28.6)	43 (32.8)
Conjunctival hyperaemia	15 (30.6)	12 (28.6)	12 (30.0)	27 (29.7)	39 (29.8)
Eyelid vellus hair changes	4 (8.2)	9 (21.4)	6 (15.0)	13 (14.3)	19 (14.5)
Eyelash changes	9 (18.4)	6 (14.3)	3 (7.5)	15 (16.5)	18 (13.7)
Eyelash thickening	6 (12.2)	5 (11.9)	2 (5.0)	11 (12.1)	13 (9.9)
Blepharal pigmentation	4 (8.2)	4 (9.5)	3 (7.5)	8 (8.8)	11 (8.4)
Eyelash hyperpigmentation	1 (2.0)	3 (7.1)	2 (5.0)	4 (4.4)	6 (4.6)
Hypertrichosis	3 (6.1)	1 (2.4)	0	4 (4.4)	4 (3.1)
Eye irritation	0	0	3 (7.5)	0	3 (2.3)
Punctate keratitis	1 (2.0)	1 (2.4)	1 (2.5)	2 (2.2)	3 (2.3)

Patients with multiple events in each row were counted only once, at the maximum severity. Group 1 included patients with baseline IOP ≥16 and <22 mmHg; Groups 2 and 3 included patients with baseline IOP ≥22 and ≤34 mmHg

^a^All ADRs shown were mild in severity. No severe ADRs were reported.

*ADR* adverse drug reaction, *AE* adverse event, *IOP* intraocular pressure, *SAE* serious adverse event

ADRs (AEs related to study treatment) were reported in 29 patients (59.2%) in Group 1, 25 (59.5%) in Group 2, and 26 (65.0%) in Group 3 (Table 3). The majority of ADRs were ocular events, most commonly eyelash growth (incidence of 22.4–42.5% across treatment groups) and conjunctival hyperaemia (28.6–30.6%). One ADR of moderate keratitis was reported in 1 patient (2.0%) receiving sepetaprost in Group 1; otherwise, all ADRs were mild in severity. Additional safety analyses showed that in patients who received sepetaprost monotherapy (i.e., Groups 1 and 2), most ocular ADRs occurred within the first 90 days of treatment (Supplementary Table 1).

## Discussion

The phase 3 ANGEL-J2 study demonstrates the potent IOP-lowering efficacy of sepetaprost, both as monotherapy and in combination with timolol, in patients with OAG or OHT. In Groups 1 and 2, once-daily treatment with sepetaprost was associated with rapid and significant reductions in mean diurnal IOP sustained over 52 weeks. Mean diurnal IOP reductions in Groups 1 and 2 were numerically smaller at week 52+1 day (approximately 36–44 hours after the last sepetaprost dose), but remained statistically significant versus baseline. Comparatively larger IOP reductions were observed in Group 3 throughout the treatment period, demonstrating an additive effect of combining once-daily sepetaprost with twice-daily timolol. Both sepetaprost monotherapy and combination treatment with timolol demonstrated a favourable safety profile; all ADRs except one were mild in severity, and no severe or serious ADRs were reported. These data suggest that dual FP/EP3 receptor activation with sepetaprost is a tolerable and efficacious treatment strategy for the long-term management of patients with OAG or OHT.

The results of this study support findings from the phase 3 ANGEL-J1 study, which compared the efficacy and safety of sepetaprost versus latanoprost in patients with POAG or OHT [27]. In ANGEL-J1, patients randomised to sepetaprost had a mean diurnal IOP of 23.3 mmHg at baseline, which was reduced by an LS mean of 5.8 mmHg at week 2 and then maintained over 3 months of treatment [27]. Similarly, Group 2 of the present study had a mean diurnal IOP of 23.2 mmHg at baseline and significant LS mean reductions of 5.9–6.5 mmHg over the first 12 weeks; however, this study additionally shows that early IOP reductions with sepetaprost were maintained through week 52 with ongoing treatment. Reductions in mean diurnal IOP from baseline were comparatively smaller in Group 1 (4.0–4.6 mmHg over 52 weeks) but were nevertheless statistically significant, thus demonstrating the IOP-lowering efficacy of sepetaprost in a patient population with lower IOP baselines and a higher prevalence of NTG. In addition, MMRM analyses show that LS mean reductions in mean diurnal IOP were greater in Group 3 versus Group 2 (7.3–7.8 mmHg over 52 weeks), highlighting the additive efficacy of combining increased uveoscleral outflow with sepetaprost and reduced aqueous humour production with timolol [29].

The most frequently reported ADRs in this study were eyelash growth and conjunctival hyperaemia, both of which are common AEs associated with prostaglandin analogue treatment for glaucoma [10, 30]. In particular, conjunctival hyperaemia was the most common ADR among patients receiving sepetaprost monotherapy (Groups 1 and 2), and the 1-year incidence of this event (29.7%) was within the range of rates reported in previous studies of tafluprost (16.2% [31]), omidenepag isopropyl (18.8% [12]), bimatoprost (44.7% [32]), and travoprost (49.5% [33]). Additional analyses in this cohort found that most conjunctival hyperaemia ADRs were reported within 30 days of initiating sepetaprost, which supports previous findings that conjunctival hyperaemia typically occurs during the early stages of prostaglandin analogue treatment [30, 34]. Moreover, most ocular ADRs in Groups 1 and 2 occurred within the first 90 days of treatment and all ocular ADRs thereafter were mild in severity; this suggests that AEs associated with sepetaprost do not tend to accumulate or worsen with longer-term treatment. Interestingly, deepening of the upper eyelid sulcus (DUES) is another AE common to prostaglandin analogues [35], but was not observed over 52 weeks in this study. Overall, all ADRs except one (and all conjunctival hyperaemia events) were mild, and no serious AEs were related to study treatment, supporting the longer-term tolerability of sepetaprost, as monotherapy or in combination with timolol, in patients with OAG or OHT.

This study was conducted in a small sample of patients in Japan (N=131) and excluded those with a history of IOP-lowering surgery; therefore, this may limit the generalisability of our findings to other regions and patient groups. In particular, only 19 patients (15%) in this study had a primary diagnosis of NTG; given that NTG is common in Japanese clinical practice [36], further studies are warranted to confirm the safety and efficacy of sepetaprost in this patient population. Although previous active-controlled studies have evaluated the comparative efficacy and safety of sepetaprost for up to 3 months of treatment [23–27], future head-to-head studies are needed to directly compare longer-term outcomes between sepetaprost and other IOP-lowering therapies. Moreover, given that glaucoma is a chronic disease that requires lifelong treatment and monitoring, further studies should assess the efficacy and safety profile of sepetaprost beyond 52 weeks of follow-up.

In conclusion, the phase 3 ANGEL-J2 study demonstrated the 1-year safety and IOP-lowering efficacy of sepetaprost, as monotherapy or in combination with timolol, in patients with OAG or OHT. Significant reductions in IOP were achieved and maintained over 52 weeks of once-daily sepetaprost monotherapy, and additive IOP-lowering effects were observed when sepetaprost was used in combination with twice-daily timolol. The results of this study suggest that sepetaprost, a novel dual agonist of FP and EP3 receptors, represents a tolerable, efficacious, and durable treatment option for the long-term management of OAG or OHT.

## Supplementary Information

Below is the link to the electronic supplementary material.

Supplementary file1 (PDF 247 KB)

## Data Availability

The datasets generated and/or analysed during the current study are not publicly available but may be requested in accordance with Santen’s data sharing policy.
