# Supplementary material for: Long-term safety and efficacy of sepetaprost, with or without timolol, in patients with open-angle glaucoma or ocular hypertension: results from the randomised, phase 3 ANGEL-J2 study
Source: Jpn J Ophthalmol. 2026 Jun 16;70(4):940–8. doi: 10.1007/s10384-026-01351-2 (PMC13452872; doi:10.1007/s10384-026-01351-2)
Supplement: Supplementary file 1 [file 10384_2026_1351_MOESM1_ESM.pdf]

Supplementary material

Long-term safety and efficacy of sepetaprost, with or without timolol, in patients with open-angle glaucoma or ocular hypertension: results from the randomised, phase 3 ANGEL-J2 study

Authors: Masaru Inatani, Yuko Tone, Toshihiro Ikeda, Raymund Angeles

Corresponding author: Raymund Angeles, Santen Inc., 2100 Powell Street, Suite 800, Emeryville, CA 94608, USA. Phone: +1-510-368-9861; E-mail: Raymund.Angeles@santen.com

Supplementary Fig. 1 ANGEL-J2 patient disposition

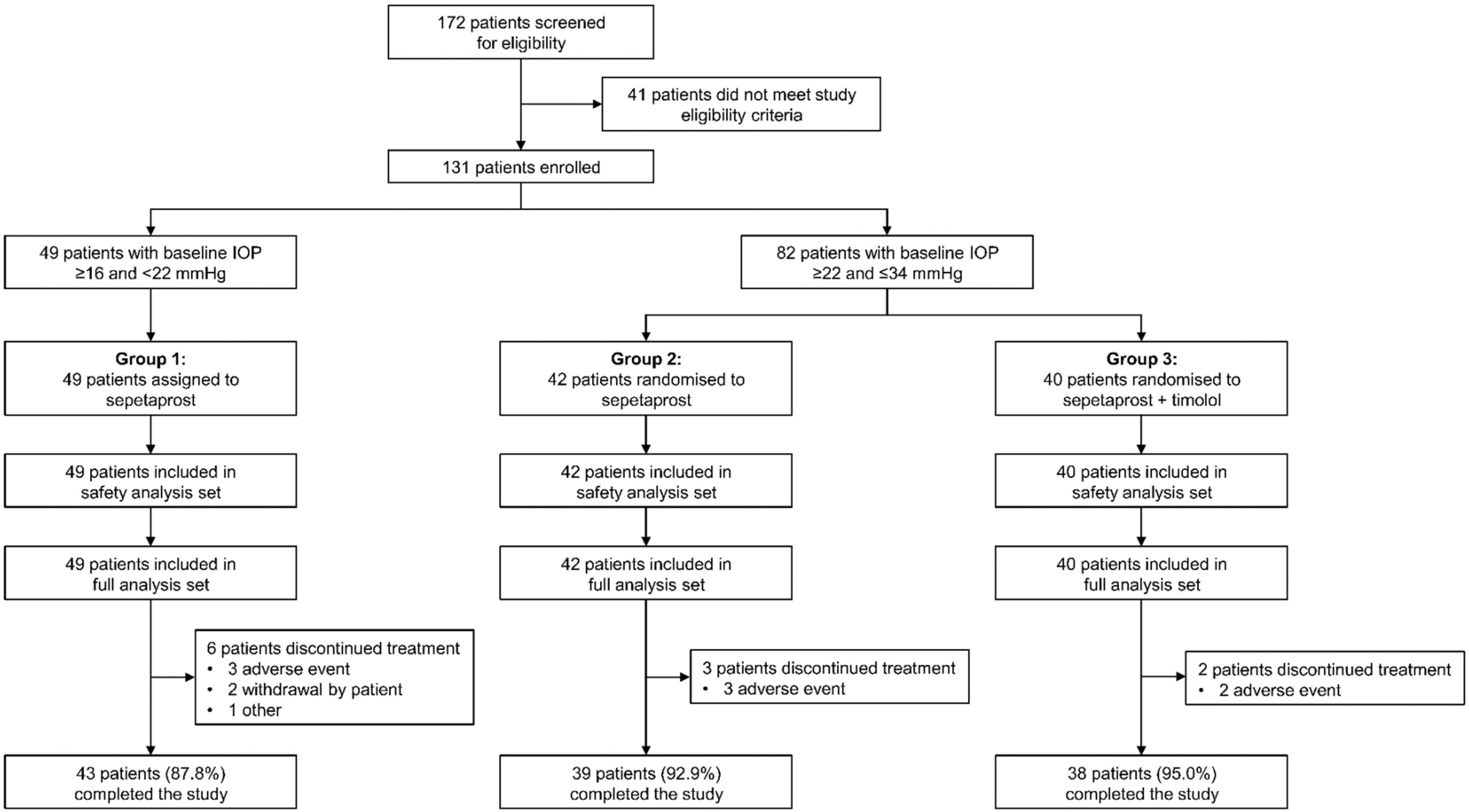

IOP, intraocular pressure.

Supplementary Table 1 Incidence of ocular and subcutaneous ADRs by time of onset in Groups 1 and 2 (safety analysis set)

	**Study period**[a]					
**n (%)**	**Day 1–30 (n=91)**	**Day 31–90 (n=90)**	**Day 91–180 (n=87)**	**Day 181–270 (n=86)**	**Day 271–360 (n=84)**	**Day >360 (n=71)**
Patients with any ADR	31 (34.1)	19 (21.1)	11 (12.6)	9 (10.5)	3 (3.6)	6 (8.5)
Eye disorders	30 (33.0)	19 (21.1)	10 (11.5)	8 (9.3)	3 (3.6)	6 (8.5)
Conjunctival hyperaemia	22 (24.2)	2 (2.2)	0	3 (3.5)	0	0
Growth of eyelashes	6 (6.6)	13 (14.4)	5 (5.7)	1 (1.2)	1 (1.2)	0
Eyelash thickening	3 (3.3)	5 (5.6)	1 (1.1)	1 (1.2)	0	1 (1.4)
Eyelash changes	2 (2.2)	6 (6.7)	2 (2.3)	2 (2.3)	1 (1.2)	2 (2.8)
Eyelash hyperpigmentation	2 (2.2)	0	0	0	1 (1.2)	1 (1.4)
Punctate keratitis	2 (2.2)	0	0	0	0	0
Blepharal pigmentation	1 (1.1)	2 (2.2)	1 (1.1)	3 (3.5)	1 (1.2)	0
Conjunctival irritation	1 (1.1)	0	0	0	0	0
Eyelid vellus hair changes	1 (1.1)	5 (5.6)	2 (2.3)	2 (2.3)	1 (1.2)	2 (2.8)
Keratitis	1 (1.1)	0	0	0	0	0
Eyelid oedema	0	1 (1.1)	0	0	0	0
Iris hyperpigmentation	0	0	0	1 (1.2)	0	0
Photopsia	0	1 (1.1)	0	0	0	0
Trichiasis	0	0	0	0	0	1 (1.4)
Trichomegaly	0	0	0	0	0	1 (1.4)
Skin and subcutaneous tissue disorders	1 (1.1)	0	3 (3.4)	0	0	0
Hypertrichosis	1 (1.1)	0	3 (3.4)	0	0	0

Patients with multiple ADRs within the same preferred term were counted only once, at the earliest incidence. Patients with multiple ADRs within the same system organ class were counted once in each study period, for the ADR with the earliest incidence.

[a]Analyses included patients with ≥1 visit in a given study period.

ADR, adverse drug reaction.
